# Temperamental and Neurocognitive predictors in Korean basketball league draft selection

**DOI:** 10.1192/j.eurpsy.2024.782

**Published:** 2024-08-27

**Authors:** B. W. Nam, D. H. Han, S. M. Kim, J. Hong, H. Hwang, K. J. Min

**Affiliations:** ^1^Dr. Nam’s Psychiatric Clinic, Choongju; ^2^Chung Ang University Hosptial, Seoul, Korea, Republic Of

## Abstract

**Introduction:**

The Korean Basketball League(KBL) holds an annual draft to allow teams to select new players, mostly graduates from the elite college basketball teams even though some are from high school teams. In sports games, many factors might influence the success of an athlete. In addition to possessing excellent physical and technical factors, success in a sports game is also influenced by remarkable psychological factors. Several studies reported that elite sports players can control their anxiety during competition, which may lead to better performance. In particular, the temperament and characteristics of players have been regarded as crucial determinants of the player’s performance and goal. In this regard, numerous studies suggest that personality is considered to be an important predictor of long-term success in professional sports

**Objectives:**

Based on previous reports and studies, we hypothesized that physical status, temperament and characteristics, and neurocognitive functions of basketball players could predict the result of KBL draft selection. Especially, temperament and characteristics were associated with the result of KBL selection. The basketball performances including average scores and average rebound were associated with emotional perception and mental rotation.

**Methods:**

We recruited the number of 44 college elite basketball players(KBL selection, n=17; Non-KBL selection, n=27), and the number of 35 age-matched healthy comparison subjects who major in sports education in college. All participants were assessed with the Temperament and Character Inventory(TCI), Sports Anxiety Scales(SAS), Beck Depression Inventory(BDI), Perceived Stress Scale (PSS-10), Trail Making Test(TMT), and Computerized Neuro-cognitive Test(CNT) for Emotional Perception and Mental Rotation.

**Results:**

Current results showed that physical status, temperament and characteristics, and Neurocognitive functions of college basketball players could predict the KBL draft selection. Among temperament and characteristics, novelty seeking and reward dependence were associated with KBL draft selection. The basketball performances including average scores and average rebound were associated with emotional perception and mental rotation.

**Conclusions:**

In order to be a good basketball player for a long time, it was confirmed that temperamental factors and Neurocognitive factors were very closely related. Furthermore, it is also judged that these results can be used as basic data to predict potential professional basketball players.

**Disclosure of Interest:**

None Declared

